# Reviews

**Published:** 1879-09

**Authors:** 


### REVIEWS.

---

**On Diseases of the Stomach; the Varieties of Dyspepsia, their Diagnosis and Treatment.** By S. O. HABERSHON, M. D., F. R. C. P., &c. Third Edition. Philadelphia: Lindsay & Blakiston. 1879.

The writer is well known as an author, a number of other works treating upon similar subjects being before the public. The present work is quite comprehensive, and contains much that proves the subject to have been thoroughly studied. It is divided into twenty chapters upon nearly as many varieties of dyspepsia. This rather extreme classification gives the impression of diffuseness, and we are inclined to think this its weakest point. Compared with a work upon a similar subject (and a model in its way), viz: Chambers' on Indigestion, it is less happy in its style and hardly equal to it in clearness of logical deductions. On the other hand, it covers a wider field and is based on broader principles.

In proof of this latter assertion, we quote a paragraph contained in Chapter III, on the General Sympathy of the Stomach in Disease. The author says: "It is the tendency of the clinical study of any isolated class of disease, or of the affections of any particular organ, to exclude the consideration of other portions of the body, as if one part could be separated from the other. The nervous system is so connected with every individual structure that it sympathizes with changes in any of them, and may be thrown into a state of general disturbance by a comparatively trifling injury. * * * With sympathies so widely pervading the whole system, it is not surprising that gastric diseases should present most varied indications in the disturbance of other organs; and, in like manner, that gastric phenomena of an abnormal kind should result from irritation, far removed from the stomach itself. The closest circumspection is required to distinguish between sympathetic affections and those of strictly local origin." V. P.

---

**Manual of the Principles and Practice of Operative Surgery.** By STEPHEN SMITH, M. D. Boston: Houghton, Osgood & Co. New York: 21 Astor Place The Riverside Press, Cambridge. 1879.

This book contains 662 pages, with numerous illustrations—733 in all—and is an enlargement of the author's hand-book of "Surgical Operations," edited 1862, including the general operations of surgery in civil practice, while the former was designed more for military practice. It contains a great deal of useful information, which is generally spread over a great many books, mentions all the latest improvements in operative surgery, and will, without doubt, be of service to the profession, as a short record of the state of surgery at the present time. The language is clear and distinct, but every subject is treated so briefly that it will scarcely be of great use to the student. It is of more value for the experienced physician, who has little time for study and less money to buy larger surgical works, but who yet wishes to know something of the progress of the science. He will, in this book, find the latest and best methods of performing any operation, from venesection to ovariotomy, and as a book of reference for him we recommend it most heartily. The illustrations are, on the whole, excellent; a great many of them, however, so reduced in size as to be almost of no use. M.

---

**A Clinical Treatise on the Diseases of the Nervous System.** By M. ROSENTHAL, Professor of Diseases of the Nervous System at Vienna. With a preface by Professor CHARCOT. Translated by L. PUTZEL, M. D. New York: William Wood & Co.

This is volume seven of Wood's Library of standard medical authors, and is the most valuable of the series. The author divides the work into three classes. Class I, includes diseases of the meninges and parenchyma of the brain. Class II, diseases of the medulla oblongata. Class III, diseases of the meninges and parenchyma of the spinal cord.

The discussion of each subject is concise and clear. The author, besides referring to the pathological anatomy, symptomatology, etiology of the diseases under consideration, illustrates with clinical notes of interesting cases, the prominent features of each, and thus gives a practical treatise of essential service to those members of the profession who are unable to examine the larger works. We commend this volume to the favorable notice of our readers. L.

---

**Pocket Therapeutics and Dose Book.** By MORSE STEWART, JR., B. A., M. D. Second Edition. Detroit, Mich.: Geo. D. Stewart. Price, Cloth, $1.00.

This little book is intended to serve as a *reminder*, to be referred to in emergencies and in cases of doubt. It is stated to contain a classification and explanation of the action of medicines; minimum and maximum doses in troy weights, with their equivalent in the metric weights; index and definition of diseases, with appropriate remedies; index of common and pharmaceutical names; classification of symptoms; poisons and their antidotes; and useful hints to the prescriber. It is small enough to be carried in the pocket, and those in need of such a companion for the purpose of refreshing their memory, will find it very convenient. D.

---

**Memoranda of Poisons.** By THOMAS H. TANNER, M. D., F. L. S. Fourth American, from the Last London Enlarged and Revised Edition. Philadelphia: Lindsay & Blakiston. Buffalo: T. H. Butler.

Dr. Tanner's writings are all thoroughly practical, and we have here a small but practically complete manual of Toxicology for the use of the practitioner and student. This new edition has been carefully revised, and much new matter added, and the typographical arrangement is such as to show at a glance the treatment to be adopted in each particular case of poisoning to which a medical man is liable to be summoned. D.

---

**Public Health.** A new weekly Journal devoted to the dissemination of Sanitary Knowledge and the Preservation of Health.

It is published by Dr. Edward I. Birmingham, the well-known editor of the *Hospital Gazette* who has secured the co-operation of some of the leading sanitarians in the country. We believe with the editor, that a weekly sanitary journal, properly conducted (and Dr. Birmingham's connection with it ensures that) will meet with sufficient encouragement and support to ensure its permanency. We wish the new journal God-speed in its effort to awaken interest in sanitary matters, and we hope there will be a demand for *Public Health* by every enlightened member of the community. D.

---

**Long Life and How to Reach It.** American Health Primers Series. Philadelphia: Lindsay & Blakiston. Buffalo: J. H. Butler.

The volume is from the pen of Joseph G. Richardson, M. D., and contains many of the plain, common-sense maxims in regard to fresh air, proper food and drink, etc., which cannot be too forcibly impressed on the minds of people at large. These primers are gotten up in neat style and compact form, and, if people will but read and properly digest their contents, they may be of use in the community. M.

